# Fast Bound Pool Fraction Mapping Using Stimulated Echoes

**DOI:** 10.1002/mrm.22846

**Published:** 2011-03-24

**Authors:** M Soellinger, C Langkammer, T Seifert-Held, F Fazekas, S Ropele

**Affiliations:** Department of Neurology, Medical University of GrazGraz, Austria

**Keywords:** STEAM, myelin, bound pool fraction, two-pool model, MRI, magnetization transfer, brain

## Abstract

Magnetization transfer imaging advanced to an indispensible tool for investigating white matter changes. Quantitative magnetization transfer imaging methods allow the determination of the bound pool fraction (BPF), which is thought to be directly linked to myelin integrity. Long acquisition times and high specific absorption rates are still inhibiting broad in vivo utilization of currently available BPF mapping techniques. Herewith, a stimulated echoes amplitude modulation-based, single-shot echo planar imaging technique for BPF and *T*_1_ quantification is presented at 3T. It allows whole brain mapping in 10–15 min and is low in specific absorption rates. The method was validated with different concentrations of bovine serum albumin (BSA) phantoms. Intra- and inter-subject variability was assessed in vivo. Phantom measurements verified linearity between bovine serum albumin concentrations and measured BPF, which was independent of *T*_1_ variations. *T*_1_ values in the phantoms correlated well with values provided by standard *T*_1_ mapping methods. Intrasubject variability was minimal and mean regional BPFs of 10 volunteers (e.g., left frontal white matter = 0.135 ± 0.003, right frontal white matter = 0.129 ± 0.006) were in line with previously published data. Assessment of interhemispheric BPF differences revealed significantly higher BPF for the left brain hemisphere. To sum up, these results suggest the proposed method useful for cross-sectional and longitudinal studies of white matter changes in the human brain. Magn Reson Med, 2011. © 2011 Wiley-Liss, Inc.

Determination of the macromolecular content in brain tissue is essential for investigating white matter (WM) changes typically found in normal ageing as well as in inflammatory and neurodegenerative diseases of the central nervous system. A prominent example is multiple sclerosis, with focal demyelination and reduction of intact myelin in normal appearing WM (1). Conventional MRI is based on relaxation properties of freely moving tissue water. Because of their ultrashort *T*_2_ relaxation times, bound protons do not contribute to the MR signal significantly. Therefore, conventional MRI exhibits only limited capability to assess microstructural information, whereas magnetization transfer imaging is sensitive to changes in WM integrity (2–4). Magnetization transfer (MT) is based on the capability of proton pools hosted by different molecular environments to exchange their magnetization by means of chemical and dipolar interactions.

In 1993, Henkelman et al. (5) described MT quantitatively (qMT) with a two-pool model, which reduces the relaxation model of brain tissue to two proton compartments, one pool of protons associated with free mobile water and a second pool consisting of protons bound to macromolecules, which are restricted in their mobility. Among the parameters describing the two-pool model, the bound pool fraction (BPF) is of particular interest. BPF is the molar fraction of protons bound to macromolecules, thus reflecting macromolecular proton density. There is convincing evidence that BPF is the pool parameter most directly linked to the composition and density of myelin (6–8).

During the last decades, different acquisition concepts for assessing some or all of the fundamental pool parameters have been developed. In gradient echo-based BPF determination schemes, off-resonant radiofrequency (RF) pulses are used for sampling the saturation profile. Pool parameters are then evaluated using different mathematical models (9–13). Gochberg et al. (14) proposed the analysis of *T*_1_ relaxation curve following on-resonant selective inversion recovery (IR) prepulses, and recent improvements in sequence design allowed first in vivo measurements in the human brain (15,16). Nevertheless, these methods are still not suitable for daily clinical use at 3T, as they are very time consuming. A low specific absorption rate (SAR) method was introduced by Lee and Dagher (17) but its clinical application was hampered by long acquisition times. Latest incorporations of MT into balanced steady-state free precession signal equations facilitate whole brain qMT imaging (18). Promising results were presented for 1.5T (19), whereas its use at higher field strengths is challenging due to high SAR levels. Additionally, shimming becomes an important issue for whole brain coverage at higher field strengths.

In 2003, Ropele et al. (20) proposed a low-SAR BPF mapping concept based on stimulated echoes amplitude modulation (STEAM). Limitations for broad clinical use were its restriction to single slice acquisition and its sensitivity to B_1_ inhomogeneities. While the latter could be reduced with composite pulses (21), overall scan time was still rather lengthy.

In this work, a STEAM-based technique with multiple mixing times (TM) is presented for BPF quantification. We demonstrate that apparent *T*_1_ and BPF can be obtained with an interleaved, multislice, single-shot echo planar imaging (sshEPI) readout scheme, which allows rapid whole brain mapping of *T*_1_ and BPF. The new approach was implemented on a clinical 3T scanner and validated in cross-linked bovine serum albumin (BSA) phantoms. For in vivo validation, repeatability was assessed by measuring a single volunteer five times subsequently. Additionally, the proposed method was used to determine apparent T_1_ and BPF in various brain regions of 10 healthy volunteers.

## THEORY

**FIG. 1:**
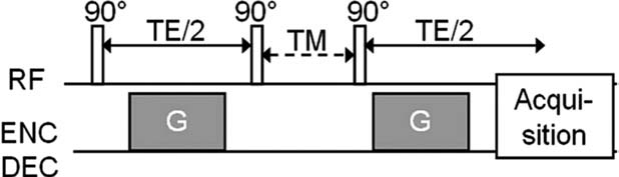
Basic STEAM experiment. A gradient (G) between the first two radiofrequency (RF) pulses impresses a modulation onto the transverse magnetization. Magnetization preparation is followed by longitudinal relaxation of the modulation during mixing time (TM). After the third RF pulse still modulated magnetization is selectively captured by applying a demodulation gradient before acquisition.

The two-pool model (5) fully describes the phenomenon of MT between two tissue compartments. For better understanding of the sequence proposed, implications of the two-pool model on the STEAM (Fig. 1) experiment are shortly outlined.

**FIG. 2:**
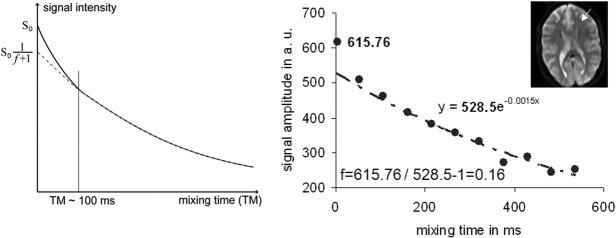
Signal behavior of a STEAM experiment of brain tissue as a function of TM, corresponding to (Eq. 9; left, continuous line). After both proton pools reach a steady state, signal decay is monoexponential, corresponding to (Eq. 10; left, dotted line). The pool size ratio *f* can be calculated from the net magnetization *S*_0_ before any MT takes place and a monoexponential fit of the signal decay rate, as here presented for frontal WM (right).

STEAM labeling is highly *T*_2_ selective. Protons with *T*_2_ being significantly smaller than echo time (TE)/2 are not labeled since their transversal magnetization decays during encoding time, or as it is for protons bound to tissue macromolecules, they are not effectively rotated by the RF pulses. Hence, only protons associated with water are labeled. Thus, at the very beginning of TM, labeled magnetization is only present in the free pool. During TM, biexponential decay of the labeling in the free pool is governed by two effects, longitudinal relaxation, and MT between the mobile and bound protons. As shown in (20) decay of the labeled magnetization *M*_f_(*t*)—the longitudinal magnetization of the labeled spin ensembles of the free water pool, by MT can be modeled using indicator dilution theory. *M*_f_(*t*) serves as an indicator, which is diluted by MT to the bound proton pool. Therefore, the MT effect is mathematically described by adding first-order transfer rates of the free (*k*_fb_) and bound pool (*k*_bf_) to longitudinal relaxation experienced by all magnetization-saturated spin ensembles. Corresponding Bloch equations for the transient longitudinal magnetization of the two pools after labeling (*M*_f_(*t*), *M*_b_(*t*)) are


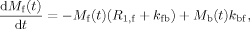
[1]


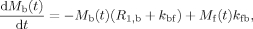
[2]

with *R*_1,f_ and *R*_1,b_ being the longitudinal relaxation rates for the free and bound proton pool, respectively. As mentioned above, magnetization labeling does not affect protons bound to tissue macromolecules because its magnetization is not effectively rotated by the first RF pulse. Even in the case of partial saturation of the bound proton pool by the RF pulse, the spins in the bound pool are not affected by the labeling because the transverse magnetization will vanish during a typical pulse separation time of a several milliseconds during magnetization preparation. Thus, the condition, that *M*_b_(*t* = 0) is negligibly small is fulfilled for all tissues with sufficiently small *T*_2b_. The succeeding RF excitation pulse in combination with a demodulation gradient directly maps the labeled magnetization onto the acquired MR signal as a function of TM (Fig. 2). The measured signal solely represents *M*_f_(*t*) as *M*_b_(*t*) decays very fast to be captured. Therefore, it is sufficient to solve Eq. 1 and Eq. 2 for *M*_f_(*t*), which results in


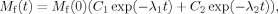
[3]

with


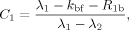
[4]


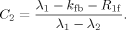
[5]

λ_1_ and λ_2_ correspond to fast decay and conventionally observed longitudinal relaxation decay, respectively. More specific, the fast decay rate λ_1_ describes the approach of steady state of the magnetization of the two pools (22). According to dilution theory (20), steady state is reached, when the labeled spin concentration is equal in both pools. Under the assumptions that *k*_bf_>*R*_1,f_, *R*_1,b_ (20,23,24) and with


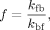
[6]

which applies for the two-pool model, constants *C*_1_ and *C*_2_ reduce to


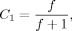
[7]


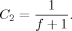
[8]

Eventually, the signal reflecting *M*_f_(*t*), *S*(*t*) results in


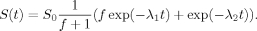
[9]

The latter equation describes the signal behavior as a function of TM (Fig. 2).

After the bound and free proton pools have approached steady state, magnetization modulation decays monoexponentially (Fig. 2), simplifying (Eq. 9) to


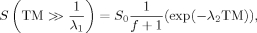
[10]

with the relaxation rate λ_2_ corresponding to *T*_1_ and *S*_0_ being the net magnetization of the free proton pool before any MT has taken place. Consequently, performing STEAM experiments with minimal TM and sampling of the monoexponential decay curve allows the determination of apparent *T*_1_ and *f*. Hence, BPF can be calculated from the pool size ratio f according to BPF ≡ *f*/(*f* + 1).

## MEASUREMENTS

### Sequence

**FIG. 3:**
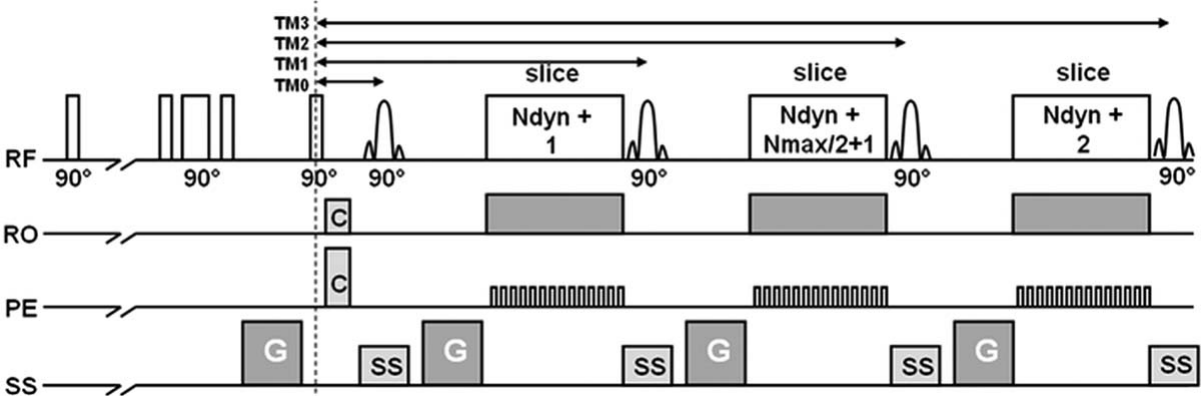
Schematic of the proposed multislice STEAM sequence. Nonslice-selective water-only preparation is followed by slicewise sshEPI readout with different TMs. The slices are permuted differently with each dynamic (*N*_dyn_), i.e., with each new steam preparation, and distance of subsequently acquired slices was half of the slice package thickness (*N*_max_/2).

A multislice, sshEPI STEAM sequence for mapping BPF was implemented on a clinical 3T scanner (Tim Trio, Siemens Healthcare, Erlangen, Germany). As depicted in Fig. 3, a nonslice-selective 90° pulse initially nulls longitudinal magnetization for subsequent experiments. A short recovery period of 2 s is followed by nonslice-selective magnetization preparation. It consists of two rectangular 90° pulses, with the first one being a binomial water-only RF pulse, enveloping a modulation gradient (G). After shortly crushing residual transverse magnetization (C), only labeled magnetization is read out with a slice-selective 90° excitation pulse followed by the demodulation gradient G and a sshEPI readout scheme. All slices are acquired subsequently, thus experiencing different TMs but identical magnetization modulation. The experiment is repeated *n* times permuting the slice order such that all TMs are measured for each slice, i.e., the number of permutations is given by the number of slices. To minimize crosstalk between adjacent slices, the acquisition scheme within one dynamic was designed in a way that slices acquired subsequently are spatially separated by half of the total slice package size. This acquisition scheme is then permuted cyclically with the number of dynamics *N*_dyn_ (Fig. 3). Sequence parameters for all measurements presented were: minimal mixing time TM_0_ = 3.2 ms, TM_*i*_ = (60 × *i*) ms + TM_0_, where *i* ranged from 1 to 10, echo time of the echo planar imaging readout (TE/2) = 22 ms, repetition time (TR) = 2600 ms, acquisition matrix = 100 × 100, spatial resolution: 2.5 × 2.5 × 5 mm^3^, number of slices *N*_max_ = 11, and number of dynamics *N*_dyn_ = 11. Ten signal averages were collected in a total acquisition time of 4.8 min. Measurements with mixing times TM_2_ to TM_10_ were used for calculating apparent *T*_1_ by a single exponential fit. TM_1_ = 63 ms was excluded to ensure steady state of MT, which is assumed to be reached after 100 ms. TM_0_ was used as an estimate for *S*_0_, which is the labeled magnetization before any MT or longitudinal relaxation take place.

### Phantom Measurements

For validation of the proposed method, a test array with 11 samples was set up. Cross-linked BSA was used as a two-pool relaxation model for human brain tissue (25). Different concentrations of cross-linked BSA mimicked varying macromolecular content in brain tissue, as BPF is expected to increase linearly with BSA concentration. Thus, linear correlation between BSA concentration and measured BPF values was analyzed on a test samples prepared as follows. A commercially available solution of 30% BSA to water per weight (Sigma-Aldrich) was diluted with water (physiological saline: 0.9% NaCl) to six different concentrations of 0.1, 0.14, 0.18, 0.22, 0.26, and 0.30% BSA to water per weight. To cross-link BSA, the samples were heated for 10 min in a water bath of 80°C. Per dilution, one plastic tube with an outer diameter of 28 mm was filled. Three further samples with BSA concentration of 0.18% BSA to water per weight were additionally doped with 0.1, 0.02, and 0.04 mmol/L Gadolinium (Gd-DTPA) to vary *T*_1_ while remaining the size of the macromolecular proton fraction unchanged. The two residual tubes were filled with water and a MnCl_2_ solution of 0.25 mmol/L; they served as references and served as references with different longitudinal relaxation rates but zero macromolecular proton fraction.

For comparison of *T*_1_ values derived from the STEAM method, *T*_1_ was calculated supplementary from a standard multislice IR turbo spin echo based (26) and a driven equilibrium single pulse observation of *T*_1_ (DESPOT1) (27) sequence. IR scans comprised five scans with different inversion times ranging from 100 to 3200 ms and spatial resolution of 1 × 1 × 4 mm^3^. DESPOT1 *T*_1_ maps were calculated from two spoiled gradient echo sequences with flip angles of 4° and 15°, TR/TE = 9.8 ms/4.77 ms and a bandwidth of 140 Hz per pixel. For comparison of the three *T*_1_ mapping approaches, mean *T*_1_ of the samples within a central slice was used.

### In Vivo Measurements

Measurement repeatability was assessed by five repetitive examinations of the same volunteer (female, 33 years old), in separate scan sessions, by the same operator on different days over 6 weeks. Thereafter, a total of 10 healthy volunteers (six males/four females, age: mean 31 years, range 27–51 years) was examined with the proposed sequence.

After reconstructing *T*_1_ and BPF maps, regions of interest (ROIs) were outlined on the *T*_1_ maps separately for each hemisphere for the head of caudate nucleus, putamen, corona radiata, frontal and occipital WM. Additionally, ROIs were identified in the splenium and genu of the corpus callosum. ROIs were compared by analysis of variance (ANOVA) for the repeated measurements as well as for the 10 different subjects examined. Interhemispheric difference of BPF was investigated by applying a two-sided Wilcoxon signed-rank test to regional BPFs derived from the 10 healthy volunteers. *P* values < 0.05 were considered statistically significant.

## RESULTS

### Phantom Measurements

**FIG. 4:**
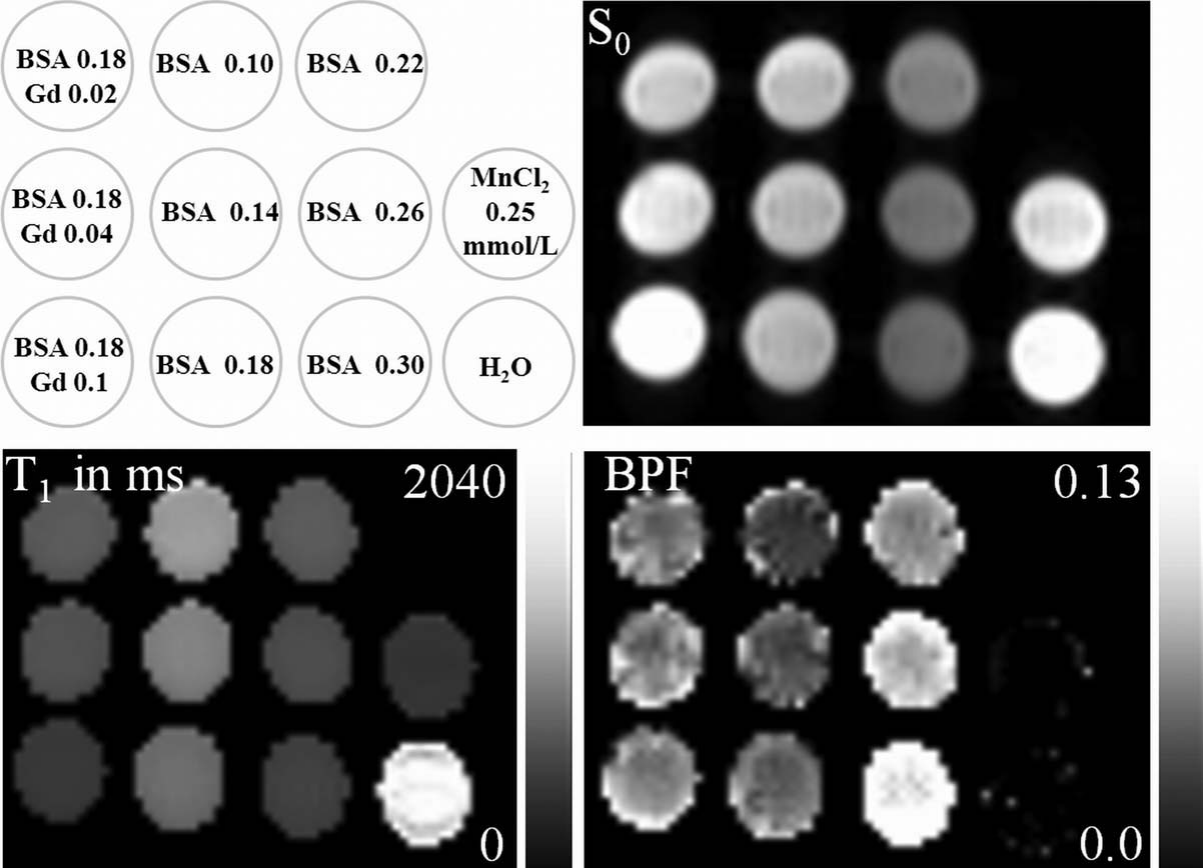
Arrangement of the test rig with phantom tubes containing different BSA, Gd, and MnCl_2_ concentrations and corresponding *S*_0_ map (above). *S*_0_ represents the magnitude image acquired with minimum TM. *T*_1_ and BPF maps are presented below. BSA concentrations are given in % BSA to water per weight, Gd concentrations in mmol/L.

**FIG. 5:**
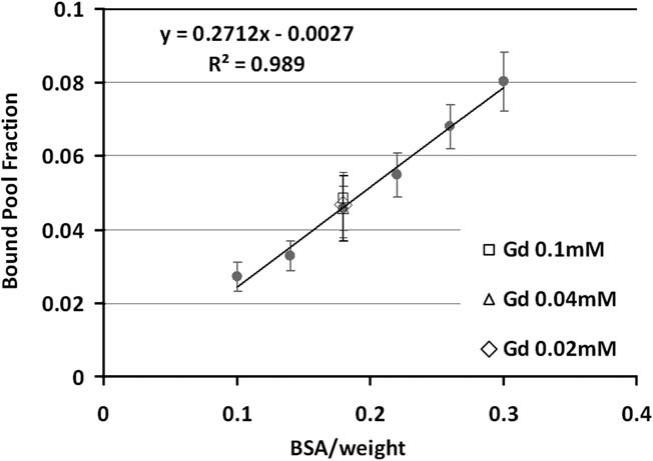
Measured bound pool fractions (BPF) as a function of different concentrations of cross-linked BSA. Three samples were doped additionally with different Gadolinium (Gd) concentrations. Error bars indicate the standard deviations of included pixels (*n* > 10).

**FIG. 6:**
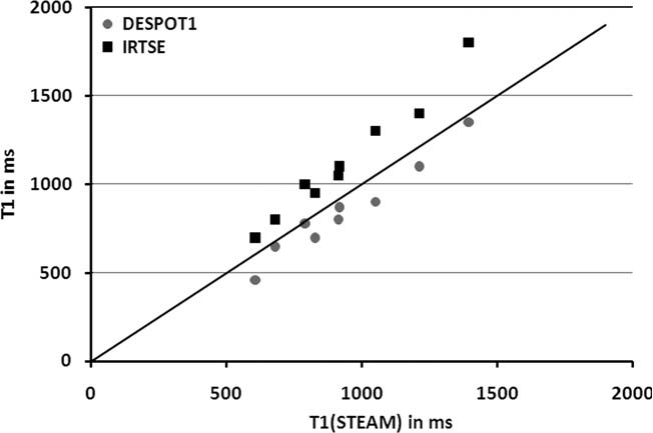
Effective *T*_1_ values of the samples acquired with the proposed method compared to *T*_1_ values acquired with an inversion recovery turbo-spin-echo-based and DESPOT1 sequences.

Phantom measurements (Fig. 4) revealed the theoretically expected linear relationship between different BSA concentrations and corresponding BPF values for all slices (Fig. 5). The offset at zero concentration was 0.0027, which is well within the standard deviation. Residual BPF values of the reference samples were 0.008 ± 0.012 and 0.009 ± 0.009 and for water and MnCl_2_, respectively. No significant difference was found between probes with identical BSA but different Gd-DTPA concentrations. *T*_1_ values of the different BSA samples determined by the STEAM approach were lower compared to the values determined by the IR method but slightly higher than data acquired with DESPOT1, as shown in Fig. 6.

### In Vivo Measurements

Before scanning a group of volunteers, repeatability was assessed by examining the same volunteer five times. Analysis of variance for repeated measurements revealed a lower variance of BPF within the ROIs of 41 × 10^−6^ compared to 30 × 10^−4^ between the different ROIs. Regional results are presented in Table 1.

**Table 1:** Mean and ±SD of *f*, BPF and T_1_ of Five Repeated Measurements in the Same Volunteer in Different White (WM) and Grey (GM) Matter Structures for the Right and Left Hemispheres

		*f*	SD	BPF	SD	*T*_1_ (ms)	SD (ms)
WM cc genu^a^		0.194	0.017	0.163	0.012	725	34
WM cc splen^b^		0.149	0.006	0.130	0.005	730	21
WM frontal	left	0.156	0.005	0.135	0.003	739	17
	right	0.149	0.007	0.129	0.006	744	16
WM occipital	left	0.149	0.005	0.129	0.004	744	10
	right	0.139	0.007	0.121	0.005	752	10
WM cor rad^c^	left	0.134	0.006	0.118	0.004	754	13
	right	0.127	0.003	0.112	0.002	746	16
GM caud nucl^d^	left	0.102	0.005	0.092	0.004	989	45
	right	0.089	0.007	0.082	0.006	998	61
GM putamen	left	0.109	0.013	0.098	0.011	991	57
	right	0.105	0.008	0.094	0.006	954	39

a corpus callosum genu;

b corpus callosum splenium;

c corona radiata;

d caudate nucleus.

**FIG. 7:**
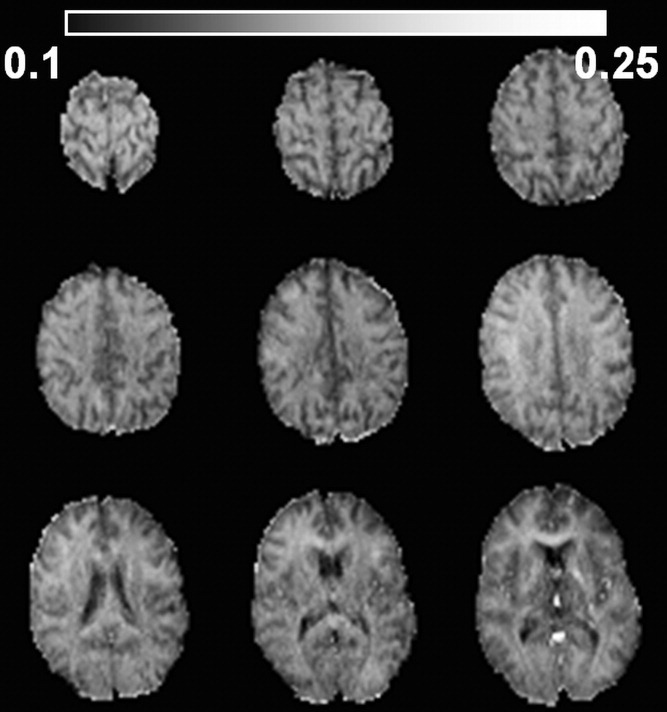
Representative BPF-maps of a 41-year-old healthy male volunteer.

In the successive feasibility study, determination of BPF maps was accomplished successfully in all 10 volunteers. Representative BPF maps from a healthy volunteer are provided in Fig. 7. Results of regional BPF and *T*_1_ values are summarized in Table 2. Highest BPF was found in the genu of the corpus callosum, while lowest BPF was detected in grey matter regions. Standard deviations of the different regions were high compared to the ranges of the repeatability study, especially in the genu of the corpus callosum and the caudate nucleus. BPF in the corpus callosum was significantly higher than in any other WM region investigated. No significant differences were found within other WM regions. Including all ROIs evaluated in both hemispheres, significantly (*P* = 0.007) higher BPF was detected in the left brain hemisphere.

**Table 2:** Mean and ±SD of *f*, BPF and T_1_ of 10 Healthy Volunteers in Different White (WM) and Grey (GM) Matter Structures for the Right and Left Hemispheres

		*f*	SD	BPF	SD	*T*_1_ (ms)	SD (ms)
WM cc genu^a^		0.22	0.02	0.18	0.03	686	65
WM cc splen^b^		0.18	0.02	0.15	0.02	668	43
WM frontal	left	0.17	0.01	0.14	0.01	687	28
	right	0.16	0.01	0.14	0.02	687	35
WM occipital	left	0.16	0.01	0.13	0.01	714	50
	right	0.15	0.01	0.13	0.01	705	40
WM cor rad^c^	left	0.15	0.02	0.13	0.01	725	35
	right	0.14	0.02	0.12	0.01	711	46
GM caud nucl^d^	left	0.13	0.03	0.11	0.02	977	71
	right	0.11	0.03	0.10	0.03	1025	72
GM putamen	left	0.12	0.01	0.11	0.02	989	80
	right	0.11	0.01	0.10	0.01	953	65

a corpus callosum genu;

b corpus callosum splenium;

c corona radiata;

d caudate nucleus.

## DISCUSSION

An MT sensitive, low SAR, stimulated echo amplitude modulation-based imaging sequence has been presented for mapping BPF in the brain. The proposed method was evaluated in vitro by using phantom tubes filled with compounds of different BSA, Gd, and MnCl_2_ concentrations. An in vivo validation study was performed to assess inter- and intra-subject variations.

In vitro experiments revealed a high linearity between BPF and BSA concentration with negligible offset residuals for zero BSA concentrations. Slightly lower BPF values compared to earlier published data (20) can possibly be accredited to imperfect cross-linking and systematic errors, which are discussed in further detail below. Gd doped phantom tubes with identical BSA concentration and *T*_1_ ranging from 600 to 1000 ms did not exhibit any significant difference in BPF, which confirms *T*_1_ insensitivity of the new STEAM method within this range. Monoexponential fitting of signals from longer TMs, necessary for BPF determination, generates the by-product of apparent *T*_1_ maps. Derived values are well within the range of values acquired with two established methods, namely IR turbo spin echo based and DESPOT1. Underestimation of STEAM-derived *T*_1_ compared to IR data is probably attributable to diffusion effects introduced by the modulation gradients, which accelerate signal decay with increasing TM. Additionally, direct saturation effects caused by excitation of proximate slices, already minimized by the alternating slice acquisition described above, might still decrease apparent *T*_1_. Compared to *T*_1_ acquired with DESPOT1, STEAM derived *T*_1_ values were slightly higher. Underestimation of DESPOT1 compared to IR turbo spin echo based likely arises from the high flip angle sensitivity at 3T, which was not corrected for (28).

The in vivo repeatability study performed with the proposed method revealed generally low variances in the investigated regions. Therefore, the method should suit well for follow-up studies investigating changes in myelin content or microstructural changes in brain tissue. BPFs observed in our cohort of healthy volunteers in frontal WM were generally in good agreement with values reported in literature, ranging from 0.13 to 0.16 (10,11,18,29). Corresponding variances were large compared to the aforementioned repeatability study, suggesting high intersubject differences in macromolecular content. Susceptibility of sshEPI to *B*_0_ inhomogeneities caused by different head geometries might additionally have increased intersubject variability. Significantly higher BPF was found in the left brain hemisphere compared to the right hemispheres, which is in agreement with earlier MTR findings (30).

For deriving quantitative parameters from images acquired subsequently, it is crucial, that all images combined for parameter determination experience the same preparation. Therefore, for each new excitation, equilibrium magnetization before magnetization manipulation needs to be independent from preceding magnetization alterations. The problem was tackled by introducing a nonfrequency selective 90° block pulse for saturation after each dynamic followed by a recovery time of 2 s. Recovery time was optimized for WM and was adjusted such that the acquisition of the same slice order in subsequent dynamics did not show any significant signal differences for WM. Longer recovery times did increase signal-to-noise-ratio (SNR) less than the square root of scan time increases for WM. In contrast, prolongation of the waiting time would increase SNR in grey matter regions, since *T*_1_ in grey matter is higher compared to WM. Generally speaking, incomplete *T*_1_ relaxation does reduce SNR but not affect BPF calculation per se, as the saturation pulse ensures for the same signal level for each tagging preparation. Multislice sshEPI was chosen for data acquisition, as it (i) allows rapid whole brain acquisition and (ii) minimizes motion effects, to which STEAM is very sensitive (31). However, in sshEPI resonance offset effects caused by *B*_0_ inhomogeneities or spatial magnetic susceptibility variations can deteriorate image quality significantly. Moreover, image quality can suffer from image blurring due to the long *T*_2_*-weighted echo planar imaging readout interval. These effects became prominent in the proximity of air cavities and in grey matter regions with low *T*_2_*, such as the globus pallidus (Fig. 7, lower right corner) and may have caused higher BPFs in the putamen and caudate nucleus than reported elsewhere (10,11,18,29). But, these are not general limitations of the proposed method. Segmented echo planar imaging and parallel imaging might allow to shorten sampling duration and echo time and thus to minimize these adverse effects, strategies, which are to be implemented and evaluated in future work.

In contrast to the originally proposed STEAM approach (20) *B*_1_ insensitivity was achieved by eliminating the inversion pulse and reducing the basic acquisition scheme to a STEAM experiment with multiple TMs. *B*_1_ errors in the STEAM experiments can lead to imperfect labeling during magnetization preparation, which reduces SNR, at which signal is reduced in the same proportion in all images contributing to BPF determination (Eq. 10). Thus, the accuracy of BPF is on principle not affected by *B*_1_ inhomogeneities. Changed sequence design did further allow for fast multislice imaging, which had been inhibited by a nonslice-selective 180° RF pulse, which was substituted by imaging decaying magnetization labeling as a function of time with an interleaved, slice selective sshEPI readout scheme. According to (Eq. 10), derivation of BPF from these measurements boils down to determining *S*_0_ and monoexponential fitting of apparent *T*_1_. Hence, BPF strongly depends on the accuracy of *S*_0_, ideally reflecting labeled magnetization before any MT has taken place, i.e., mixing time TM_0_ equals zero. With the current implementation, shortest achievable TM_0_ was 3.2 ms. Monoexponential fitting is only valid if solely signals are used from TMs, which are long enough, so that the residual signal of the fast decay, the first term of Eq. 9, is negligibly small. Fast decay time constants 1/λ_1_ derived from qMT values at 3T in normal brain tissue (29) range from 45 to 32 ms for the investigated regions, and 29 ms are reported for lesions associated with multiple sclerosis. Therefore, for normal brain tissue, signals arising from the fast exchange term were decayed to less than 7% of its original contribution for TMs longer than 120 ms, as chosen in the presented work. Additionally, this residual signal contribution is multiplied by *f* (Eq 9; in which *f* is in the range of 0.1) in contrast to the signal associated with the slow relaxation, thus the influence of the fast decay term can be neglected, which allows for Eq. 10. qMT imaging is of particular interest in demyelinating diseases, and 1/λ_1_ is expected to be within the limits of normal brain tissue or further reduced (see above). Supplementary, *f* is reduced, and, therefore, further reduction of residual influence from fast exchange is expected for demyelinating pathologies compared with normal brain tissue. Compromises of accuracy due to the theoretical approximations that the fast magnetization exchange after 120 ms is negligible and that signal acquired with TM = 3.2 ms corresponds to signal of TM = 0 ms were simulated using Eq. 9 and literature values (29) of f, λ_1_ and λ_2_, the slow exchange rate. For simulations with the proposed method, *f* was underestimated for all regions. The effect was mostly pronounced in the caudate nucleus with a decrease of 11%. Considering the negligible influence of the fast exchange term discussed above, these BPF errors can mainly be attributed to underestimations of *S*_0_. Underestimation of BPF is in agreement with the lower BPF values in the BSA phantoms compared to the values found with the original STEAM approach (20) but contradicts the rather high BPFs found in vivo (29,32). Generally, simulations with literature values have to be treated with care, as literature values of qMT values and two-pool relaxation constants do vary significantly (29,32,33). Disadvantageously, the suggested use of stimulated echoes introduces, additionally to its intrinsically low SNR, an adverse noise bias due to inhomogeneous excitation, *T*_2_* and *T*_1_, as already addressed above. Low SNR in the images used for monoexponential fitting can introduce overestimation of the apparent longitudinal relaxation time *T*_1_ and consequently leads to overestimation of BPF, which might explain the rather high BPF values found for all in vivo data but especially in grey matter, as already discussed above.

Major advantages of this new STEAM approach compared with other methods are its speed, its truly stand-alone nature, i.e., there are no reference scans required, its simplicity and robustness of the fitting involved, and its low SAR level. First, using sshEPI in an interleaved slice excitation manner allows whole brain acquisitions in 10 to 15 min with the proposed method. It is additionally beneficial for clinical applicability that the new method, in contrast to pulsed saturation qMT (9–13) and balanced steady-state free precession (18) approaches, goes without any *T*_1_, *T*_2_ or *B*_1_ reference measurements, similar to IR-based approaches (16). In contrast to very similar IR methods, a sensitivity reduction of the new STEAM-based method by roughly a factor of 2 and motion sensitivity are traded for fast and low SAR multislice acquisition and a simple fitting procedure, enabled by the assumption of *M*_b_(*t* = 0) = 0. Monoexponential fitting of only two unknowns is very fast and robust, permitting inline BPF mapping on scanner hardware, which is highly desired for clinical routine. Additionally, SAR intense MT preparation pulses (9–13,34) are not needed, and the applied sshEPI readout scheme is low in RF power deposition compared to selective IR-fast spin echo (16) or balanced steady-state free precession (18) sequences. To our knowledge, solely STEAM-based BPF determination does not depend on any assumption of the macromolecular proton lineshape or any other pool parameter, except *T*_2b_. Yet, no value has to be assumed for *T*_2b_, the only assumption drawn is that *T*_2b_ is short enough to ensure selective free proton pool magnetization labeling and acquisition.

## CONCLUSIONS

A stand-alone, stimulated echo-based, multislice approach enabling whole brain BPF and apparent *T*_1_ mapping within 10–15 min has been proposed for 3T. In addition to in vitro verification, a study assessing repeatability and intersubject variations was conducted. Low variations in repeated measurements suggest the use of the proposed method for longitudinal observations of myelin integrity in the brain. The sequence exhibits a low SAR level and thus can be used at even higher field strengths.
